# Enhanced Range and Endurance Evaluation of a Camber Morphing Wing Aircraft

**DOI:** 10.3390/biomimetics8010034

**Published:** 2023-01-13

**Authors:** Bruce W. Jo, Tuba Majid

**Affiliations:** 1 Advanced Dynamics, Mechatronics and Collaborative Robotics (ADAMS) Laboratory, Department of Mechanical Engineering, Tennessee Technological University, Cookeville, TN 38505, USA; 2 Department of Mechanical and Process Engineering, ETH Zürich, 8092 Zürich, Switzerland

**Keywords:** camber morphing, morphing aircraft, conventional, fixed wing, flight range, flight endurance, single Re, Reynolds number

## Abstract

Flight range, endurance, maneuverability, and agility are the key elements that determine an aircraft’s performance. Both conventional and morphing wing aircraft have been well studied and estimated in all aspects of performance. When considering the performance of morphing aircraft, most works address aspects of the aerodynamical performance such as L and D as well as flight envelopes for flight dynamics and control perspectives. However, the actual benefits of adopting morphing technologies in practical aspects such as aircraft operation, mission planning, and sustainability have not been addressed so far. Thus, this paper addresses the practical aspect of the benefits when adopting a camber morphing wing aircraft. Identical geometrical and computational conditions were applied to an already-existing aircraft: the RQ-7a Shadow. The wing structure was switched between a fixed wing and a camber morphing wing to generate conventional and morphing wing geometries. The fixed-wing cases had varying flap deflection angles, and the camber morphing wing cases had varying camber rates from 4% to 8%. Once the $C_{L}$ values of the fixed and morphing wing cases were matched up to two significant figures, the $C_{D}$ and $C_{L}/C_{D}$ were analyzed for these matching cases to calculate the flight endurance, range, and improvement. When NACA 6410 is adopted, a 17% improvement in flight range and endurance average was expected. In the case of NACA 8410, an average 60% improvement was expected.

## 1. Introduction

The word ‘morph’ comes from the Greek word ‘metamorphosis’, as in ‘to change shape and form’—a biological development process in which forms change [1,2,3,4]. In engineering, morphing is a discipline of smart structures or an either compliant or rigid body mechanism-based approach. It is associated with providing systems with additional capabilities to adapt to external changes [5]. In engineering, morphing technologies refer to sustainable technologies that enhance flight performance, including maneuverability and the flight envelope, by actively adjusting aircraft wing shapes that correspond to different flight modes [6]. Whereas conventional aircraft adjust wing shapes using discrete and deployable control surfaces such as flaps, ailerons, and rudders, morphing wing aircraft use an internal morphing mechanism, thereby possibly generating more substantial wing-shaped changes toward optimal shapes [7,8,9,10,11,12,13,14,15].

Additional advantages of the absence of jointed control surfaces that conventional aircraft have include reduced assembly complexities and costs [13,16,17,18,19,20,21] and aerodynamical performance aspects. Morphing aircraft have also been verified to have improved roll control motions, a reduced $C_{D}$, an increased range, an expanded operational envelope, reduced fuel consumption and airframe noise, etc. [22,23,24,25,26,27,28,29,30,31].

The research and development of morphing technologies span almost all engineering topics and approaches. In wing design itself, some major topics include (1) design for shape optimization [32,33,34,35,36], (2) design for aerodynamic optimization [37,38,39], (3) design for aero-structure optimization [40,41,42], and (4) manufacturing process development [43,44,45]. In the focus of morphing mechanism development, topics such as (1) material selection and analysis [46,47,48,49,50,51], (2) structure and solid mechanics development [50,52,53,54,55,56,57,58,59], (3) smart materials for actuation [60,61,62,63,64,65,66,67], and (4) conventional actuators for morphing [61,66,67] are primary. In morphing wing analysis regarding design and implementation, (1) analytical or experimental modeling, (2) validation [43,68,69,70,71], (3) performance and characteristics analysis [72,73,74,75,76,77], and (4) aeroelasticity analysis [78,79,80] are the topics being investigated. Other than those previously mentioned, control systems and flight control and dynamics for morphing wing aircraft [81,82,83] are also seriously studied. In the sense of the technology readiness level (TRL) of morphing aircraft, various levels of analysis and validation have been performed that range from analytical and numerical works to large-scale wind tunnel and flight tests [43,71,84]. Emphasizing manufacturability and feasibility according to implementation aspects, skin structure design and analysis of morphing wings is probably one of the most popular studied subjects [85,86,87,88].

Among many topics, this paper relates to morphing wing analysis regarding design and implementation topic (3)—performance and characteristics analysis of morphing wing aircraft [89,90,91]. Moreover, among the many types of morphing, this paper mainly targets camber morphing in the airfoil.

## 2. Motivation

The performance of an aircraft is determined by the key elements: flight range, endurance, maneuverability, and agility [92]. Conventional and morphing wing aircraft have been studied and estimated [93,94]. Studies that investigated the performance of morphing aircraft addressed aerodynamical performance aspects such as L and D as well as flight envelopes for flight dynamics and control [27,31,90,91,95,96,97,98,99]. Some representative works of a camber morphing wing aircraft’s aerodynamical characteristics compared their performance to those of conventional wing aircraft [72,73]. However, the flight range and endurance of a camber morphing wing aircraft have been overlooked.

Another motivation for this work regarded a more practical aspect of research on morphing wings and aircraft toward realization in commercial or military lines. Unfortunately, most of the literature is not based on a practical aircraft model, which implies a lack of practicability and feasibility for actual flight. However, we began from an already existing UAV model with a fixed wing. We then switched to the same UAV model with a camber morphing wing with the same geometric parameters (chord length, etc.), specifications, and operational conditions. This was done to investigate the benefits of morphing wing aircraft regarding enhanced flight range and endurance. The flight range and endurance are directly related to energy efficiencies, fuel consumption, and sustainability for futuristic applications.

**Figure 1:**
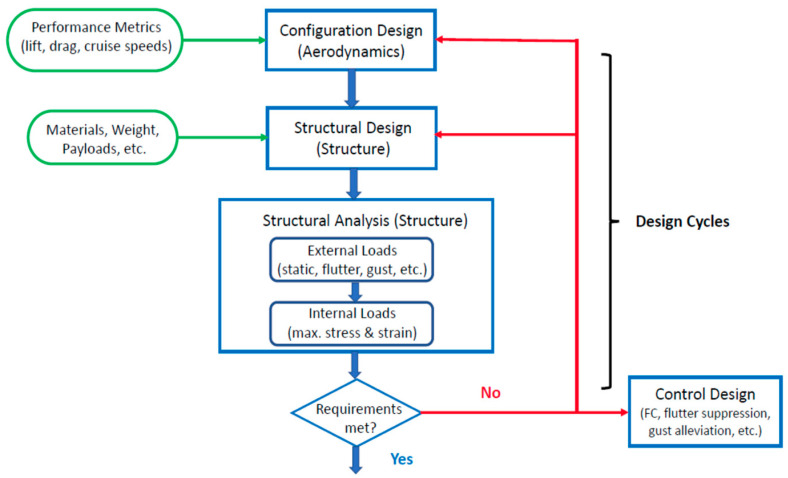
Flowchart of aircraft design and analysis cycle (image courtesy of J.T. Kim).

In the aircraft design process, the first step is to configure the aerodynamical performance metrics, including the flight ranges and endurances. Thus, it is crucial to precisely understand and acknowledge the actual benefits of flight while adopting morphing wings instead of conventional fixed wings. The flowchart shown in Figure 1 explains the aircraft design cycle.

## 3. Problem Statement

This paper analyzes and explains the expected benefits of a morphing wing aircraft through a comparative study with conventional, fixed-wing aircraft under identical conditions. To emphasize the aspect of real-world applications, we adopted an already-existing UAV model and operational conditions. The flight range and endurance are the two elements that determine flight performance, and they are functions of L, D, or L/D. To maintain and guarantee a precise analysis, we matched L on cases of both a camber morphing wing and a fixed wing under identical geometrical and aerodynamical conditions. Then, we analyzed differentiations of D, and the flight range and endurance were analyzed using D.

Thus, the main objectives of this work were: (1) to match *L* with the flap-deflection angle from a fixed wing and a camber rate from a morphing wing; and then (2) to compare L, D, and L/D between these two types of wings. The differentiated flight ranges and endurance from both cases were computed under the same operational conditions.

## 4. Computational Model

Computational models for morphing and conventional wings were constructed to satisfy identical geometries. Then, a computational analysis using ANSYS FLUENT was adopted to match *L* values to determine differences in their *L*, *D*, and *L/D*. Table 1 below shows the operational parameters and conditions that were applied to both models to determine the Re, speed, and other external conditions. As categorized in the review of modeling and analysis of morphing wings [70], linear and non-linear methods are used for computing steady-state aerodynamics. Linear methods are better suited for thin lifting surfaces such as an airfoil at a small AoA. Since this study was conducted over a range of AoAs that included higher AoAs, the CFD method was chosen over linear methods.

The CFD simulation software ANSYS FLUENT was employed with the RANS-based Spalart–Allmaras (SA) turbulence model. A C-type unstructured mesh was used in the FLUENT setup in which the dimensions of the fluid domain were set based on the recommended length [100]. First, the FLUENT setup was benchmarked. Then, the settings were maintained as identical to those of a previous study described in [73]. The values for the air density (μ) and velocity (v) inputs corresponded to the cruising conditions of our RQ-7a Shadow UAV model as shown in Table 1.

For the wall boundary conditions, ANSYS FLUENT has a built-in enhanced wall treatment (EWT) feature available in its Spalart–Allmaras model that automatically blends all solution variables from their viscous sublayer formulation to the corresponding logarithmic layer values depending on $y^{+}$ as shown in Equation (1):

(1) $$\frac{u}{u_{\tau }}=\frac{\rho u_{\tau }y}{\mu }=\frac{1}{\kappa }lnE\left(\frac{\rho u_{\tau }y}{\mu }\right)\;$$

where *u* is the velocity parallel to the wall, $u_{\tau }$ is the shear velocity, *y* is the distance from the wall, κ is the von Kármán constant (0.4187), and *E* = 9.793.

However, it should be noted that EWT alone is not sufficient to generate wall $y^{+}$ values of less than one at the airfoil boundary, so inflation layers had to be generated to resolve the viscous sublayer in the near-wall region. A total of 25 inflation layers were generated around the airfoil with a growth rate of 1.1 to resolve the viscous sublayer in the near-wall region. The first layer thickness (*y*) was computed using Equation (2) to maintain a $y^{+}$ value of less than one at the wall:

(2) $$y=\frac{y^{+}µ\;}{\rho \sqrt{\frac{1}{2}C_{f}v^{2}}}$$

where µ is the dynamic viscosity, ρ is the air density, and v is the cruise velocity as shown in Table 1.

The skin friction $C_{f}$ was calculated using Equation (3):

(3) $$C_{f}=0.058\;Re^{-0.2}$$

The AoA (α) was made a parameter in the FLUENT setup, and the value of α varied from 0° to 15°. The L and D computed by ANSYS ($F_{X}$ and $F_{Y}$) were further manipulated using Equation (4):

(4) $$\left[\begin{array}{c} \;L\;\\ \;D\; \end{array}\right]=\left[\begin{array}{cc} \;\;\;\;cos\;\alpha & sin\;\alpha \;\\ -sin\;\alpha \; & cos\;\alpha \; \end{array}\right]\left[\begin{array}{c} \;F_{X}\;\\ \;F_{Y}\; \end{array}\right]$$

FLUENT does not have the option to set the direction vector of *L* and *D* as a parameter, so the normal *x* and *y* components obtained for each α ($F_{X}$ and $F_{Y}$) had to be transformed.

Various morphed and deflected geometries were generated to mimic the behavior of a morphing wing and a conventional wing with a deflecting flap. These airfoil geometries were drawn in Fusion 360 and imported into the ANSYS Workbench Design Modeler in step format.

## 5. Methodology

The UAV model (RQ-7 Shadow) used a NACA 2410 airfoil with a chord length of 0.54 m. Correspondingly, the morphed configurations were generated by varying the camber rate (the first digit of the four-digit NACA series) in increments of 2% to generate three configurations: NACA 4410, NACA 6410, and NACA 8410. In contrast, the conventional airfoil configurations were generated by rotating the trailing edge flap clockwise at the joint. The flap joint rested at 0.7c from the leading edge of the baseline airfoil (NACA 2410) with a chord length of 0.54 m. The value of the flap or deflection angle was set by matching the L generated by the morphed configurations.

For these matching L morphing and conventional airfoils, the D values were also computed and compared. The aerodynamic parameter of interest was L/D or $C_{L}$*/*$C_{D}$, which in aeronautics is used synonymously with aerodynamic performance. L/D or $C_{L}$*/*$C_{D}$ measures the aerodynamic cruising efficiency and can affect the fuel consumption, range, endurance, etc. The Breguet range equation is written as:

(5) $$R=\left(\;\frac{v}{c}\;\right)\left(\;\frac{L}{D}\;\right)ln\left(\;\frac{W_{i}+W_{fuel}}{W_{i}}\;\right)\;$$

where *R* is the cruise segment range, v is the constant cruise speed, $W_{fuel}$ is the fuel weight, $W_{i}$ is the fixed weight of the aircraft, and c is the thrust-specific fuel consumption for specific operating conditions. When taking other terms in Equation (5) to be constant, the maximum range can be achieved by maximizing the quantity $C_{L}$*/*$C_{D}$ on a particular cruise operation.

Similarly, based on Equation (5), to minimize the environmental impact of fuel consumption and reduce the carbon emission rate, the amount of fuel used should be as small as possible; by looking strictly at the aerodynamics, the quantity $C_{L}/C_{D}$ should be maximized in order to minimize the amount of fuel burned.

The endurance $t_{e}$ of an aircraft can be calculated as range divided by speed, which is denoted as:

(6) $$t_{e}\left(s\right)=\left(\;\frac{\eta }{c}\;\right)\left(\;\frac{C_{L}{}^{1.5}}{C_{D}}\;\right)\sqrt{\frac{2\rho S}{W_{i}}}\;\left[\sqrt{\left(\;\frac{W_{i}+W_{fuel}}{W_{i}}\;\right)}-1\right]$$

where η is the propulsion system efficiency and *S* is the wing planform area. The endurance of an aircraft is a function of its power output; thus, the total propulsive power must be minimized to maximize the endurance. Assuming the specific fuel consumption is nearly constant in Equation (2), the fuel flow per unit time and the $C_{D}$ must be minimized to maximize the endurance, which can be achieved when $C_{L}{}^{1.5}/C_{D}$ (the endurance factor) is maximized across all points of the mission.

For airfoil cases, four camber rates were analyzed: NACA 2410 was used for the baseline; and NACA 4410, NACA 6410, and NACA 8410, were used for comparison.

## 6. Results

**Figure 2:**
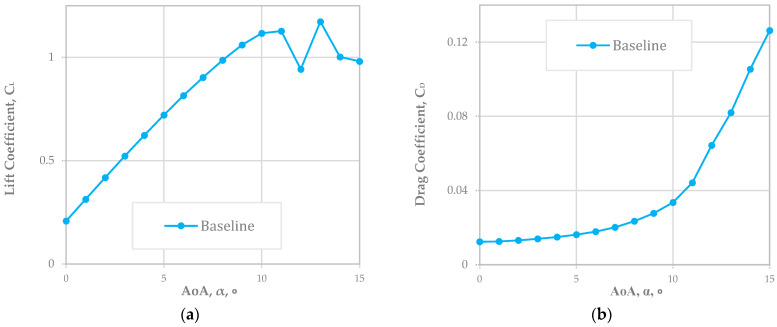
(**a**) $C_{L}$ and (**b**) $C_{D}$ plots of baseline NACA 2410 airfoil (Re = 778,179).

Figure 2 below shows the case of NACA 2410 with the baseline of the $C_{L}$ and $C_{D}$ varying the AoA by up to 15°. As noted, both the $C_{L}$ and $C_{L}$ increased as the AoA increased, and the stall angle was around 11°. The operational condition was Re at 778,179 for the RQ-7a Shadow. Through this, we confirmed the that computational models were sufficiently accurate to run other cases and that the $C_{L}$ and $C_{D}$ were reliable compared to the benchmark data [72,73,77].

Once the computational model for the airfoil was verified, the simulations for comparison were run for the three morphed configurations when the AoA changed from 0 to 15°. The $C_{L}$ morphed configurations were adequately matched against the conventional airfoil using flap-angle variations and by computing the *L* values of deflected configurations at each AoA to match the L values. The relationships between the camber rate and the flap-deflection angle are also presented in Table 2, Table 3 and Table 4. Additionally, Table 2, Table 3 and Table 4 present data for the $C_{L}/C_{D}$ and for the endurance ratio ($C_{L}{}^{1.5}/C_{D}$).

**Figure 3:**
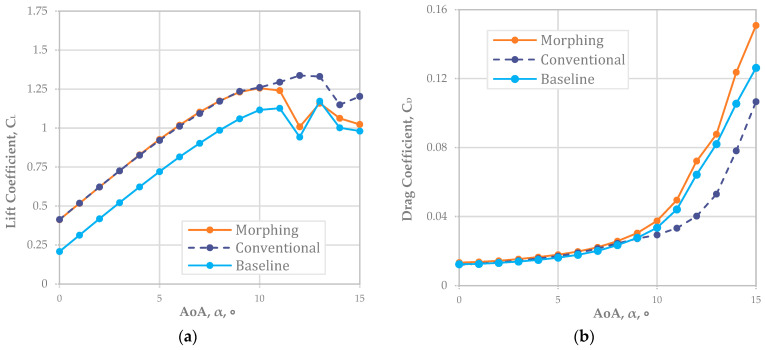

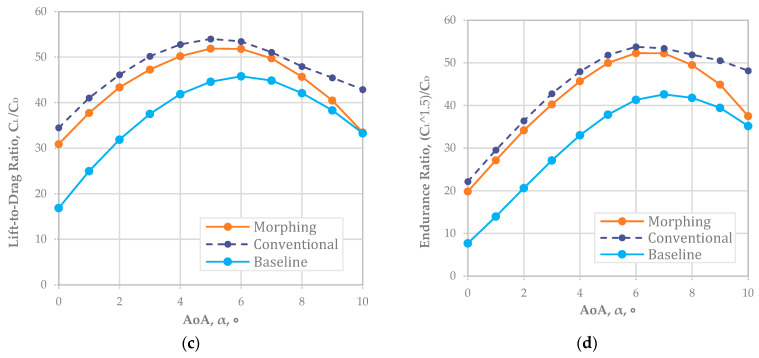
Matching *L* morphing (NACA 4410) and conventional airfoil plots (Re = 778,179): (**a**) L plot; (**b**) *D* plot; (**c**) *L/D*; (**d**) endurance ratio.

For NACA 4410, Figure 3a shows the $C_{L}$ was well matched for both the conventional and morphing wings. The offset value of about 0.21 in $C_{L}$ implied that the NACA 4410 had a 0.21 greater $C_{L}$ than NACA 2410 (or a 2% higher camber rate was around a 0.21 $C_{L}$ increment). Based on the graph in Figure 3a, the reliable AoA was up to 10° because the stall began right after 10°. As shown in Figure 3b, we analyzed $C_{D}$; the results implied that a camber morphing wing at a 4% camber rate (NACA 4410) had an almost identical $C_{D}$ to that of the conventional fixed wing. However, the graph separation began around 7° for the AoA when the $C_{D}$ of the fixed wing was less than that of the camber morphing wing.

Figure 3c,d are directly related to $C_{L}$, $C_{D}$, and $C_{L}/C_{D}$. The $C_{L}/C_{D}$ indicated the improvement in the flight range; Figure 3d does so for the endurance improvement. We noted that conventional fixed-wing aircraft still had a better flight range and endurance when the NACA 4410 airfoil was adopted.

The improvement rate of the flight range was maximized to about 11.5% at a 2° AoA and minimized to about 2.7% at a 9° AoA. On average, an approximately 5.9% improvement in the flight range was expected when NACA 4410—the baseline airfoil—adopted a deflecting flap rather than a camber morphing wing.

In endurance, the improvement rate was maximized to about 11.7% at a 2° AOA and minimized to about 2.25% at a 9° AoA. An average of about a 5.8% improvement in the flight endurance was expected with NACA 2410 with a deflecting flap adopted rather than a 2% camber morphed wing (NACA 4410).

**Figure 4:**
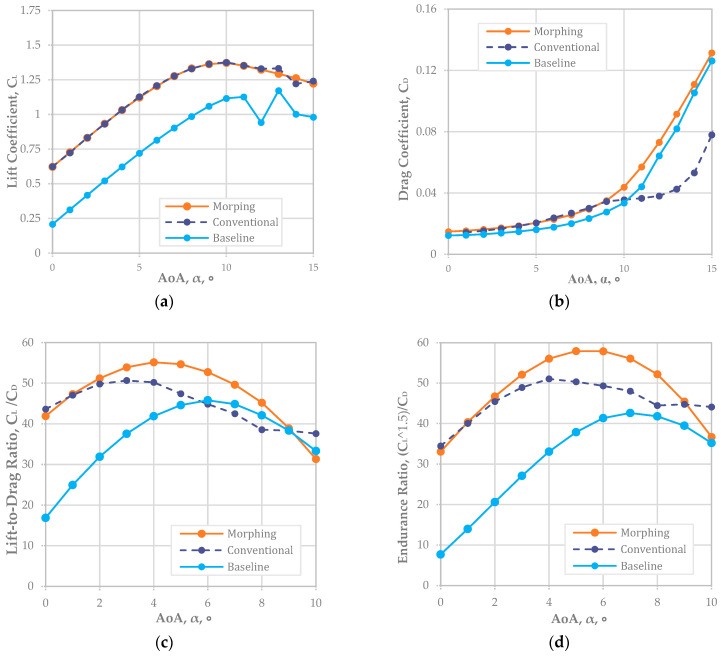
Matching $C_{L}$ morphing (NACA 6410) and conventional airfoil plots (Re = 778,179): (**a**)$\;C_{L}$; (**b**)$\;C_{D}$; (**c**)$\;C_{L}/C_{D}$; (**d**) endurance ratio.

For NACA 6410, Figure 4a shows the $C_{L}$ was well matched for both the conventional and morphing wings. The offset value of about 0.5 in $C_{L}$ implied that the NACA 6410 had a 0.5 greater $C_{L}$ than NACA 2410 (or a 4% higher camber rate was around a 0.5 $C_{L}$ increment). Based on the graph in Figure 4a, the reliable AoA was up to 10° because the airfoil began to stall after 10°. As shown in Figure 4b, we analyzed $C_{D}$; the results implied that a camber morphing wing at a 6% camber rate (NACA 6410) had an almost identical $C_{D}$ to that of the conventional fixed wing. However, the graph began to separate at an AoA of around 5° when the $C_{D}$ of the camber morphing wing was less than that of the fixed wing.

Similarly, $C_{L}$ and $C_{D}$ graphs are as shown in Figure 4a,b, an improvement in the flight range $C_{L}/C_{D}$ in Figure 4c, and endurance improvement in Figure 4d. In addition, we noted that the camber morphing wing aircraft had a better flight range and endurance when the NACA 6410 airfoil was adopted.

The improvement rate of the flight range was maximized to about 17.6% at an 8-degree AoA and was minimized to about 0.5% at a 1° AoA. On average, an approximately 4.7% extension in the flight range was expected with a 6% camber morphed wing compared to a fixed wing with a deflecting flap.

The endurance factor maximized to about 17.4% at an 8° AoA and minimized to about 0.85% at a 2° AoA. This resulted in about a 7.8% improvement in the flight endurance with a morphing wing with a NACA 6410 airfoil rather than a fixed-wing airfoil with a NACA 2410 baseline configuration with a deflecting flap.

**Figure 5:**
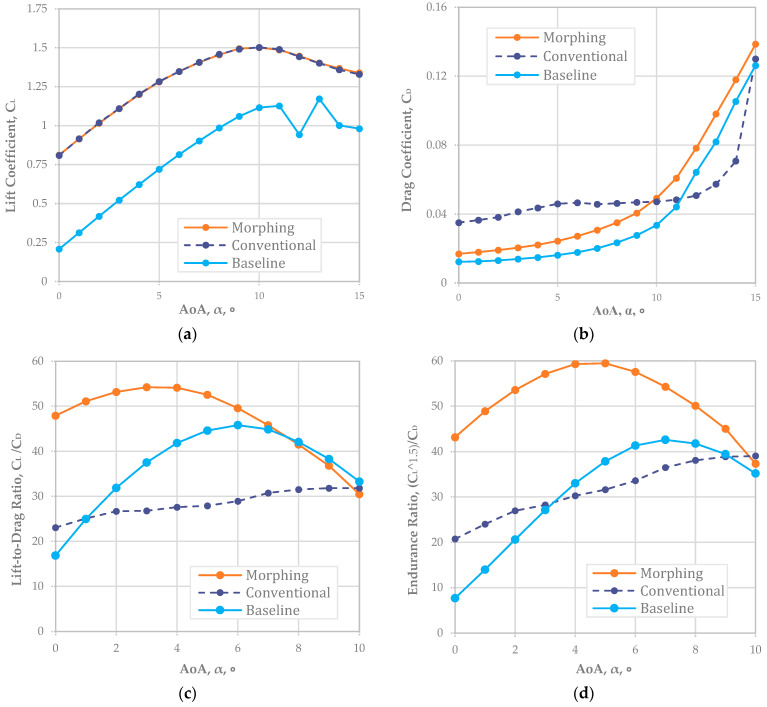
Matching $C_{L}$ morphing (NACA 8410) and conventional airfoil plots (Re = 778,179): (**a**)$\;C_{L}$; (**b**)$\;C_{D}$; (**c**)$\;C_{L}/C_{D}$; (**d**) endurance ratio.

For NACA 8410, Figure 5a shows the $C_{L}$ was well matched for both the conventional and morphing wings, with an offset of about 0.6 against the baseline airfoil that implies that the NACA 8410 airfoil has a 0.6 greater $C_{L}$ than NACA 2410 (or a 6% higher camber rate was around a 0.6 $C_{L}$ increment). Based on the graph in Figure 5a, the reliable AoA is up to 10° because the stall began right after 10°. As shown in Figure 5b, we analyzed $C_{D}$; the results implied that a camber morphing wing at an 8% camber rate (NACA 8410) had an almost identical $C_{D}$ to that of the conventional fixed wing. However, the graph separation began at 0° of the AoA when the $C_{D}$ of the camber morphing wing was much less than that of the fixed wing.

As shown in Table 4, the improvement rate of the flight range was observed in the entire range of AoAs with a large number. On average, an approximately 60% improvement in the flight range was expected when the morphing wing with a NACA 8410 airfoil was adopted rather than a fixed-wing NACA 2410 baseline airfoil with a deflecting flap.

Regarding the endurance, the improvement rate was expected in the entire range of AoAs with a large number. An average improvement of approximately 61% in the flight endurance was expected when the morphing wing with a NACA 8410 airfoil was adopted.

Table 5 and Table 6 summarize all the results above with an additional improvement percentage in the flight range and endurance. For NACA 4410, the improvement rates were negative, which implied that the conventional fixed wing performed better in the flight range and endurance; however, other cases (NACA 6410 and NACA 8410) showed improvement rates and performed substantially better in terms of the flight range and endurance in the entire range of AoAs.

## 7. Summary

The NACA 2410 (the baseline of the $C_{L}$ and $C_{D}$) when varying the AoA by up to 15° was verified. Both the $C_{L}$ and $C_{L}$ increased as the AoA increased, and the stall angle was around 11°. Based on this, we confirmed that the computational models were sufficiently accurate to run other cases and that the $C_{L}$ and $C_{D}$ were reliable compared to the benchmark data.

For NACA 4410, the $C_{L}$ was well matched for both the conventional and morphing wings. The offset value of about 0.25 in the $C_{L}$ implied that the NACA 4410 has a 0.25 greater $C_{L}$ than NACA 2410 (or a 2% higher camber rate was around a 0.25 $C_{L}$ increment). We also analyzed $C_{D}$; the results implied that a camber morphing wing at a 4% camber rate (NACA 4410) had an almost identical $C_{D}$ to that of the conventional fixed wing. We noted that the conventional fixed-wing aircraft still had a better flight range and endurance when the NACA 4410 airfoil was adopted. The improvement rate of the flight range was maximized at about 11.5% at a 2° AoA and was minimized at about 2.7% at a 9° AoA. On average, an approximately 5.9% improvement in the flight range was expected when NACA 4410 with flap deflection was adopted rather than a morphing wing. Regarding endurance, the improvement rate was maximized at about 11.7% at a 2° AoA and minimized to about 2.25% at a 9° AoA. On average, an approximately 5.8% flight endurance improvement was expected when NACA 4410 with flap deflection is adopted rather than a morphing wing.

For NACA 6410, the offset value of about 0.5 in $C_{L}$ implied that the NACA 6410 has an 0.5 larger $C_{L}$ than NACA 2410 (or a 4% higher camber rate was around a 0.5 $C_{L}$ increment). We also analyzed $C_{D}$; the results implied that a camber morphing wing at a 6% camber rate (NACA 6410) had an almost identical $C_{D}$ to that of the conventional fixed wing. However, the graph separation began around a 5° AoA when the $C_{D}$ of the camber morphing wing was less than that of the fixed wing. We noted that the camber morphing wing aircraft had a better flight range and endurance when the NACA 6410 airfoil was adopted. The improvement rate of the flight range was maximized at about 17.6% at an 8-degree AoA and minimized by about 0.5% at a 1° AoA. On average, about a 4.7% higher flight range improvement was expected when the morphing wing with a NACA 6410 airfoil was adopted rather than a NACA 6410 fixed-wing airfoil with flap deflection. In endurance, the improvement rate was maximized at about 17.4% at an 8° AoA and minimized to about 0.85% at a 2° AoA. On average, an approximately 7.8% higher flight endurance improvement was expected when the morphing wing with a NACA 6410 airfoil was adopted rather than a fixed wing with a NACA 6410 airfoil with flap deflection.

For NACA 8410, the offset value of about 0.7 in $C_{L}$ implied that the NACA 8410 had a 0.7 larger $C_{L}$ larger than NACA 2410 (or a 6% higher camber rate was around a 0.7 $C_{L}$ increment). We also analyzed $C_{D}$; the results implied that the camber morphing wing at an 8% camber rate (NACA 8410) had an almost identical $C_{D}$ to that of the conventional fixed wing. We noted that the camber morphing wing aircraft had a better flight range and endurance when the NACA 8410 airfoil was adopted. The improvement rate of the flight range was observed in the entire range of AoAs with a large number. On average, an approximately 60% higher flight range improvement was expected when the morphing wing with a NACA 8410 airfoil was adopted rather than the fixed wing with a NACA 8410 airfoil with flap deflection. In endurance, the improvement rate was expected in the entire range of AoAs with a large number. On average, an approximately 61% higher flight endurance improvement was expected when the morphing wing with a NACA 8410 airfoil was adopted rather than a fixed wing with a NACA 8410 airfoil with flap deflection.

## 8. Conclusions

This paper addressed the practical aspects of adopting a camber morphing wing aircraft compared to a conventional fixed-wing aircraft. Identical geometrical and computational conditions were applied to an already-existing aircraft model: the RQ-7a Shadow. The model’s original wing structure was a NACA 2410 airfoil geometry with a trailing edge flap. In this study, the deflection angle of the flap was changed to generate the different geometric configurations of conventional wings; whereas for the morphing wing configurations, NACA 4410, NACA 6410, and NACA 8410 airfoil geometries with the same chord length were used. The corresponding $C_{L}$ matching cases were identified along with the $C_{D}$ and $C_{L}/C_{D}$; these were analyzed to calculate any improvements in flight endurance and range. Morphing the camber by 2% from the baseline airfoil (NACA 2410 to NACA 4410) did not improve the aircraft’s performance; the conventional wing with a deflecting flap performed better. However, morphing by 4% and 6% from the baseline airfoil; i.e., using NACA 6410 or NACA 8410 configurations, improvements in the flight range and endurance ratio were seen compared to conventional ones. When NACA 6410 was adopted, an average 17% improvement in the flight range and endurance was expected. By morphing 6% to generate the NACA 8410 configuration, an average improvement of 60% was seen compared to the conventional aircraft wing design. The camber morphing wing aircraft contributed mainly to the reduced $C_{D}$ compared to the fixed-wing aircraft, which could eventually increase the fuel efficiency and sustainability. The improvements seen in the computed lift-to-drag and endurance ratios supported this.

## Figures and Tables

**Table 1:** UAV model and flight specification.

	UAV Model	RQ-7 Shadow
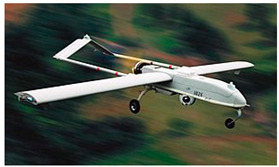	Wing chord	0.54 m
	Wingspan	3.89 m
	Cruising speeds	24.7 m/s
	Operating altitude	2000 m
	Density	1.007 kg/m m^3^
	Dynamic viscosity	1.726 × 10^−5^ Ns/m
	Re	778,179
	Baseline airfoil	NACA 2410

**Table 2:** Morphing (NACA 4410) and matching L conventional airfoils.

AoA (°)	Morphing Airfoil (NACA 4410)				Conventional Airfoils				
	$C_{L}$	$C_{D}$	$\frac{C_{L}}{C_{D}}$	$\frac{C_{L}{}^{1.5}}{C_{D}}$	$C_{L}$	$C_{D}$	$\frac{C_{L}}{C_{D}}$	$\frac{C_{L}{}^{1.5}}{C_{D}}$	Flap Angle (°)
0	0.41	0.0133	30.92	19.85	0.41	0.0120	34.49	22.18	4
1	0.52	0.0137	37.75	27.15	0.52	0.0126	41.04	29.56	4
2	0.62	0.0143	43.36	34.20	0.62	0.0135	46.14	36.41	4
3	0.73	0.0153	47.27	40.26	0.73	0.0145	50.20	42.77	4
4	0.83	0.0165	50.20	45.69	0.83	0.0156	52.79	47.96	4
5	0.93	0.0179	51.88	49.97	0.92	0.0171	54.00	51.82	4
6	1.02	0.0197	51.80	52.28	1.01	0.0189	53.45	53.76	4
7	1.10	0.0222	49.73	52.22	1.09	0.0214	51.06	53.40	4
8	1.17	0.0257	45.68	49.49	1.17	0.0244	47.95	51.90	4
9	1.23	0.0304	40.48	44.91	1.24	0.0272	45.47	50.54	3.7
10	1.26	0.0375	33.47	37.51	1.26	0.0294	42.89	48.16	2.3

**Table 3:** Aerodynamic data of morphing (NACA 6410) and matching $C_{L}$ conventional airfoils.

AoA (°)	Morphing Airfoil (NACA 6410)				Conventional Airfoils				
	$C_{L}$	$C_{D}$	$\frac{C_{L}}{C_{D}}$	$\frac{C_{L}{}^{1.5}}{C_{D}}$	$C_{L}$	$C_{D}$	$\frac{C_{L}}{C_{D}}$	$\frac{C_{L}{}^{1.5}}{C_{D}}$	Flap Angle (°)
0	0.62	0.0148	41.90	33.04	0.62	0.0143	43.61	34.45	8
1	0.73	0.0154	47.32	40.36	0.72	0.0154	47.07	40.02	8.1
2	0.83	0.0162	51.21	46.70	0.83	0.0167	49.80	45.44	8.4
3	0.93	0.0173	53.91	52.09	0.93	0.0184	50.67	48.93	8.6
4	1.03	0.0187	55.17	56.03	1.03	0.0206	50.21	51.04	8.95
5	1.12	0.0205	54.68	57.92	1.13	0.0238	47.39	50.33	9.6
6	1.20	0.0228	52.74	57.88	1.21	0.0270	44.84	49.30	9.8
7	1.28	0.0257	49.63	56.08	1.28	0.0301	42.49	48.02	9.8
8	1.33	0.0295	45.21	52.18	1.33	0.0345	38.54	44.45	9.5
9	1.36	0.0350	38.90	45.42	0.62	0.0356	43.61	44.74	8
10	1.37	0.0438	31.32	36.68	0.72	0.0366	47.07	44.07	6.1

**Table 4:** Aerodynamic data of morphing (NACA 8410) and matching $C_{L}$ conventional airfoils.

AoA (°)	Morphing Airfoil (NACA 8410)				Conventional Airfoils				
	$C_{L}$	$C_{D}$	$\frac{C_{L}}{C_{D}}$	$\frac{C_{L}{}^{1.5}}{C_{D}}$	$C_{L}$	$C_{D}$	$\frac{C_{L}}{C_{D}}$	$\frac{C_{L}{}^{1.5}}{C_{D}}$	Flap Angle (°)
0	0.81	0.0169	47.89	43.14	0.81	0.0351	23.07	20.75	17.6
1	0.92	0.0179	51.08	48.88	0.92	0.0365	25.09	24.01	17.75
2	1.02	0.0191	53.15	53.55	1.02	0.0382	26.70	26.96	18.05
3	1.11	0.0205	54.21	57.10	1.11	0.0414	26.81	28.25	18.4
4	1.20	0.0222	54.10	59.27	1.20	0.0436	27.60	30.28	18.4
5	1.28	0.0244	52.53	59.45	1.28	0.0460	27.92	31.63	18.3
6	1.35	0.0272	49.56	57.55	1.35	0.0466	28.94	33.59	18
7	1.41	0.0307	45.77	54.27	1.41	0.0457	30.76	36.49	16.2
8	1.46	0.0351	41.51	50.07	1.46	0.0463	31.53	38.08	15
9	1.49	0.0406	36.81	45.00	1.49	0.0468	31.84	38.88	13.5
10	1.50	0.0492	30.48	37.35	1.50	0.0472	31.85	39.04	11.1

**Table 5:** Comparison of the L/D or $C_{L}/C_{D}$ of morphing and conventional airfoils.

AoA (°)	Baseline	NACA 4410	Conventional	Percentage Improvement	NACA 6410	Conventional	Percentage Improvement	NACA 8410	Conventional	Percentage Improvement
0	16.86	30.92	34.49	−10.4%	41.90	43.61	−3.9%	47.89	23.07	107.6%
1	24.97	37.75	41.04	−8.0%	47.32	47.07	0.5%	51.08	25.09	103.6%
2	31.89	43.36	46.14	−6.0%	51.21	49.80	2.8%	53.15	26.70	99.0%
3	37.54	47.27	50.20	−5.8%	53.91	50.67	6.4%	54.21	26.81	102.2%
4	41.87	50.20	52.79	−4.9%	55.17	50.21	9.9%	54.10	27.60	96.0%
5	44.59	51.88	54.00	−3.9%	54.68	47.39	15.4%	52.53	27.92	88.2%
6	45.80	51.80	53.45	−3.1%	52.74	44.84	17.6%	49.56	28.94	71.3%
7	44.85	49.73	51.06	−2.6%	49.63	42.49	16.8%	45.77	30.76	48.8%
8	42.09	45.68	47.95	−4.7%	45.21	38.54	17.3%	41.51	31.53	31.6%
9	38.32	40.48	45.47	−11.0%	38.90	38.30	1.6%	36.81	31.84	15.6%
10	33.30	33.47	42.89	−22.0%	31.32	37.58	−16.7%	30.48	31.85	−4.3%

**Table 6:** Comparison of the endurance ratio of morphing and conventional airfoils.

AoA (°)	Baseline	NACA 4410	Conventional	Percentage Improvement	NACA 6410	Conventional	Percentage Improvement	NACA 8410	Conventional	Percentage Improvement
0	7.69	19.85	22.18	−10.5%	33.04	34.45	−4.1%	43.14	20.75	107.9%
1	13.97	27.15	29.56	−8.2%	40.36	40.02	0.8%	48.88	24.01	103.5%
2	20.62	34.20	36.41	−6.1%	46.70	45.44	2.8%	53.55	26.96	98.6%
3	27.12	40.26	42.77	−5.9%	52.09	48.93	6.5%	57.10	28.25	102.1%
4	33.04	45.69	47.96	−4.7%	56.03	51.04	9.8%	59.27	30.28	95.8%
5	37.85	49.97	51.82	−3.6%	57.92	50.33	15.1%	59.45	31.63	88.0%
6	41.34	52.28	53.76	−2.8%	57.88	49.30	17.4%	57.55	33.59	71.3%
7	42.61	52.22	53.40	−2.2%	56.08	48.02	16.8%	54.27	36.49	48.7%
8	41.79	49.49	51.90	−4.6%	52.18	44.45	17.4%	50.07	38.08	31.5%
9	39.45	44.91	50.54	−11.1%	45.42	44.74	1.5%	45.00	38.88	15.7%
10	35.18	37.51	48.16	−22.1%	36.68	44.07	−16.8%	37.35	39.04	−4.3%

## Data Availability

Not applicable.

## Abbreviations

**Nomenclature**	
*L*	Lift force
*D*	Drag force
$C_{L}$	Lift coefficient
$C_{D}$	Drag coefficient
AoA	Angle of attack
$C_{f}$	Skin friction coefficient
y	Distance between the airfoil wall and the first layer
Re	Reynolds number
v	Cruise velocity
Greek Symbols	
*μ*	Dynamic viscosity
*ρ*	Density of air
$u_{\tau }$	Shear velocity
κ	von Kármán constant
**Abbreviations**	
CFD	Computational fluid dynamics
RANS	Reynolds-averaged Navier–Stokes
EWT	Enhanced wall treatment
NACA	National Advisory Committee for Aeronautics
